# Supplementary material for: Identification of photoperiod-induced specific miRNAs in the adrenal glands of Sunite sheep (Ovis aries)
Source: Front Vet Sci. 2022 Jul 22;9:888207. doi: 10.3389/fvets.2022.888207 (PMC9354845; doi:10.3389/fvets.2022.888207)
Supplement: Supplementary file 1 [file Data_Sheet_1.ZIP › Supplementary file/miRDeep2 file.zip!/miRDeep2 file/Novel_Structure/LP42A1/Novel_165.pdf]

Provisional ID	:	NC_019458.2_1673
Score total	:	1.7
Score for star read(s)	:	-1.3
Score for read counts	:	0
Score for mfe	:	1.4
Score for randfold	:	1.6
Score for cons. seed	:	
Total read count	:	14
Mature read count	:	14
Loop read count	:	0
Star read count	:	0

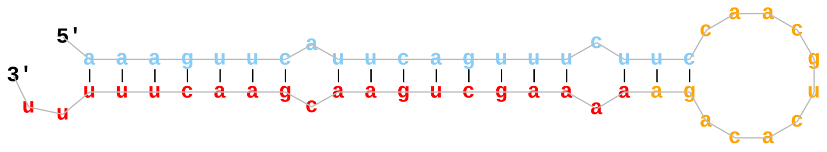

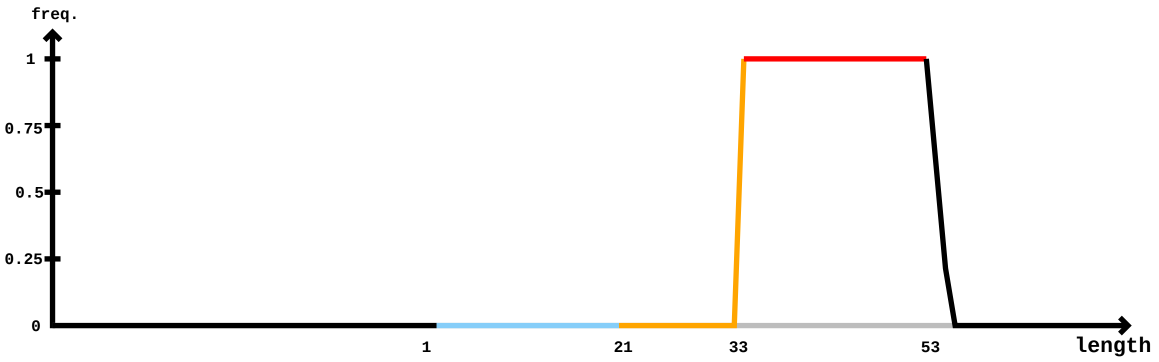

Star Mature

```
5'- auguuuccaugucuuggcuacuguaucgaguugaccaaaaaguucauucaguuucuuccaacgucacagaaaaagcugaacgaacuuuuuggccagccugguaaauaaug  -3'   exp
    ..((..(((.....)))..))..(((((.((((.(((((((((((.((((((((.(((..........))).)))))))).))))))))))).)))).))))).......   reads   mm   sample
    ......................................................................aaaaUcugaacgaacuuuuu....................   8       1    seq
    ......................................................................aaaagcugaacgaacuuuuu....................   1       0    seq
    ......................................................................aaaaCcugaacgaacuuuuug...................   2       1    seq
    ......................................................................aaaaUcugaacgaacuuuuugg..................   2       1    seq
    ......................................................................aaaagcugaacgaacuuuuugg..................   1       0    seq
```
